# Potential Roles of microRNAs in the Regulation of Monoamine Oxidase A in the Brain

**DOI:** 10.3389/fnmol.2018.00339

**Published:** 2018-09-14

**Authors:** Yuki Higuchi, Tomoko Soga, Ishwar S. Parhar

**Affiliations:** Brain Research Institute, Jeffrey Cheah School of Medicine and Health Sciences, Monash University Malaysia, Bandar Sunway, Malaysia

**Keywords:** depression, MAO, 5-HT, serotonin, non-coding RNA, miRNA, SIRT1

## Abstract

Monoamine oxidase A (MAO-A) is an enzyme that regulates the levels of monoamine neurotransmitters, such as serotonin, noradrenaline and dopamine and it has been used as a therapeutic target for depression. However, MAO-A inhibitors, which directly acts on MAO-A protein, have limited use due to their adverse effects. microRNAs (miRNAs) are 18–22 nucleotide long, small non-coding RNAs, which have recently emerged as regulators of protein levels that could potentially be new therapeutic targets for psychiatric disorders. This review article aims to discuss the current status of the treatment for depression with MAO-A inhibitors and the regulatory factors of MAO-A. Further, the review also proposes possible regulatory mechanisms of MAO-A by miRNAs, which leads to better understanding of the pathology of depressive disorders and their potential use as therapeutic agents.

## Introduction

Depression is the most prevalent mental disorder worldwide (Ferrari et al., 2013; Kessler and Bromet, 2013). The 5-hydroxytryptamine (5-HT) system, which includes the 5-HT transporters, 5-HT receptors and monoamine oxidase A (MAO-A), has been used as a drug target in the pharmacotherapeutics for depression. MAO-A inhibitors have been used as antidepressants and found to be effective especially for treatment-resistant depression and atypical depression. However, the use of MAO-A inhibitors has adverse effects on peripheral organs and causes excessive activity in the central nervous system. Therefore, identifying a novel method to inhibit MAO-A that would have no adverse effects or less adverse effect could improve the quality of medication for depression. In this review article, we address a question; Can microRNAs (miRNAs) be prominent therapeutic targets for depression by regulating MAO-A in the brain?

Patients with depression show high MAO-A levels in brain regions, such as the prefrontal cortex, midbrain and hippocampus (Meyer et al., 2006). Positron emission tomography (PET) for MAO-A in the brain shows that higher MAO-A density in the brain might contribute to the recurrence of depressive symptoms (Meyer et al., 2009), suggesting that inhibition of MAO-A may be a prominent therapy to prevent the recurrence of depression. MAO-A inhibitors act directly on MAO-A protein, inhibit its catalytic activity and result in elevated 5-HT concentration in the brain (reviewed in Finberg, 2014; Finberg and Rabey, 2016; Fišar, 2016). PET studies showed that treatment with MAO-A inhibitor, including moclobemide and phenelzine, led to extensive MAO-A blockade across brain regions (Ginovart et al., 2006; Sacher et al., 2011; Chiuccariello et al., 2015). Whole body imaging showed widespread distribution of MAO-A inhibitor, clorgyline, in peripheral organs, such as the thyroid, lung, heart and kidney (Fowler et al., 2004), indicating that action of MAO-A inhibitors is not brain-specific. Inhibition of MAO-A is effective in the treatment of atypical depression and treatment-resistant depression. Several lines of evidence suggested that MAO inhibitors are effective for tricyclic antidepressant-resistant depression (Thase et al., 1992; McGrath et al., 1993). A meta-analysis showed that MAO inhibitors are more effective for atypical depression, compared to tricyclic antidepressants (Henkel et al., 2006). For example, an MAO inhibitor, phenelzine, better prevents the recurrence of depressive symptoms, compared to tricyclic antidepressants, nortriptyline (Georgotas et al., 1989). In addition to changing neurotransmitter levels in the brain, inhibition of MAO leads to a neuroprotective effect against glucocorticoid (GC)-induced apoptosis (Johnson et al., 2010; Lam et al., 2016). M30, an MAO inhibitor with iron-chelating antioxidant properties, prevents corticosterone-induced alteration in the hippocampus, such as activation of indoleamine 2,3-dioxygenase, hippocampal apoptosis, loss of synaptic proteins and neurodegeneration and neuroinflammation (Lam et al., 2016). Selective MAO-A inhibition with pirlindole abolishes the alteration induced by chronic mild stress, such as behavioral changes in forced swimming test and the dendritic atrophy of granule neurons, but promotes adult neurogenesis in the hippocampus of rats exposed to chronic mild stress (Morais et al., 2014), indicating the effectiveness of MAO-A inhibition for stress-induced neurobiological alterations in the brain. These clinical and animal studies support the importance of proper regulation of MAO-A for treatment of depression.

The use of MAO-A inhibitors, however, has been limited because of their adverse effects. A cardiovascular effect called the “cheese effect” is caused by the inhibition of metabolism of food tyramine following MAO inhibitor treatment (Horwitz et al., 1964; Anderson et al., 1993). Under normal condition, dietary tyramine is metabolized in the gut and liver, and thus tyramine does not enter the systemic circulation. However, because MAO-A is a major enzyme which catalyzes the metabolism of tyramine, inhibition of MAO-A leads to the elevation of blood tyramine levels, followed by the potentiation of the sympathomimetic effect of tyramine, causing the elevation of blood pressure (Youdim and Bakhle, 2006). This adverse effect limits the use of MAO inhibitors for clinical treatment of depression, regardless of their effectiveness. Serotonin syndrome or serotonin toxicity is another concern in the use of MAO inhibitors. Excessive MAO-A inhibition leads to elevated 5-HT levels, which cause autonomic hyperactivity (fever, diaphoresis and tachycardia), neuromuscular hyperactivity (tremor, clonus, myoclonus and hyperreflexia), and altered mental status (Sun-Edelstein et al., 2008). Irreversible MAO inhibitors, such as tranylcypromine, can cause severe serotonin syndrome even when used alone (Boyer and Shannon, 2005). Reversible MAO-A inhibitors have also been associated with serotonin syndrome (Hawley et al., 1996a,b; Mason et al., 2000). Moclobemide causes serotonin syndrome at a higher percentage (approximately 50%) when used with other serotonergic agents, including selective serotonin reuptake inhibitors and serotonin–noradrenaline reuptake inhibitor (Isbister et al., 2003). These adverse effects of MAO-A inhibitors are mainly caused by their off-target effects in the peripheral organs and the brain. Therefore, development of methods for targeted inhibition of MAO-A in brain regions involved in the pathophysiology of depression would be necessary for the improvement of pharmacotherapy for depression. Modulating the regulators of MAO-A, such as miRNAs, is one of the possible methods for brain-specific or brain region-specific inhibition of MAO-A.

Studies have suggested that MAO-A in the brain is regulated by several factors, including transcription factors, steroid hormones and enzymes (reviewed in Higuchi et al., 2017). Recent studies suggest transcription factors for MAO-A gene, such as Kruppel like factor 11 (KLF11) and cell division cycle associated 7 like (CDCA7L; also known as R1), inhibit MAO-A in the brain (Johnson et al., 2011; Grunewald et al., 2012; Harris et al., 2015). In addition, silent mating type information regulation 2 homolog 1 (SIRT1) affects the regulation of MAO-A transcription by deacetylating helix-loop-helix transcription factor (nescient helix-loop-helix 2 (NHLH2); Libert et al., 2011) and Forkhead box O-1 (FOXO1; Wu and Shih, 2011). Furthermore, GC increases the gene expression of MAO-A through binding of GC receptors (GRs) to a GC response element in the promoter region of MAO-A gene (Ou et al., 2006). Ring finger protein 180 (RNF180), also known as ring finger protein in neural stem cells (RINES), ubiquitinates MAO-A and promotes its degradation (Kabayama et al., 2013). These regulatory factors could be potential therapeutic targets to inhibit MAO-A gene expression and miRNAs targeting their genes could also be used to modulate MAO-A in the brain.

## Potential miRNAs That Change Brain MAO-A Levels

miRNAs are small non-coding RNAs with approximately 18–22 nucleotides, which downregulate the translation of mRNAs or promote the degradation of mRNAs by binding to specific complementary sequences of target mRNAs. miRNAs play an important role in various neurobiological processes, including neurogenesis (Lang and Shi, 2012; Schouten et al., 2012), stress response (Manakov et al., 2012) and neurodegeneration (Gascon and Gao, 2012). Many miRNAs are highly conserved throughout evolution from invertebrate to vertebrate species (Davis et al., 2015). Several studies addressed the roles of miRNAs in the regulation of MAO-A. MAO-A gene is predicted to be a target of many miRNAs in miRNA target prediction (e.g., miRanda (Betel et al., 2010), TargetScan (Agarwal et al., 2015) and microT-CDS (Reczko et al., 2012; Paraskevopoulou et al., 2013)). In addition, some studies suggest that miRNAs indirectly regulate MAO-A by targeting mRNA coding the regulatory factors of MAO-A expression.

### miR-142

miR-142 family is mitochondria-enriched miRNAs in the hippocampus. In the hippocampus of rats, miR-142-3p and -5p are present at higher levels in mitochondria, compared to cytosol. miR-142-3p and -5p are detected in primary cortical neuronal, astrocyte and microglial culture prepared from rat pups; however, their levels are higher in astrocytes and microglia, compared to neuronal cells (Wang et al., 2015). This study (Wang et al., 2015) did not investigate the relationship between miR-142 family and the regulation of MAO-A. However, it would be interesting topic because the hippocampus receives serotonergic projections extensively from the median raphe nucleus (Morin and Meyer-Bernstein, 1999) and MAO-A is localized in the outer mitochondrial membrane (Edmondson et al., 2005). A few studies have shown the involvement of miR-142 in the regulation of MAO-A expression in the brain. miR-142 expression in human neuronal cell line leads to a decrease in MAO-A mRNA and enzyme activity (Chaudhuri et al., 2013a). In a prediction based on the nucleotide sequence of 3’-UTR, MAO-A mRNA is not a direct target of miR-142. However, overexpression of pre-miR-142 and transfection of miR-142-5p mimic in HEK293T cells reduces Sirtuin 1 (SIRT1) protein levels, whereas inhibition of miR-142-5p increases SIRT1 protein levels. Human neurons transduced with miR-142-expressing lentivirus also show a decrease in SIRT1 protein levels (Chaudhuri et al., 2013b). Furthermore, Chaudhuri et al. (2013a) tested the effect of SIRT1 overexpression on MAO-A protein levels in miR-142-overexpressing cells and observed that a reduction of MAO-A induced by miR-142 overexpression was abolished by SIRT1 overexpression, indicating that the effect of miR-142 on MAO-A is mediated by SIRT1.

### miR-132

**Figure 1:**
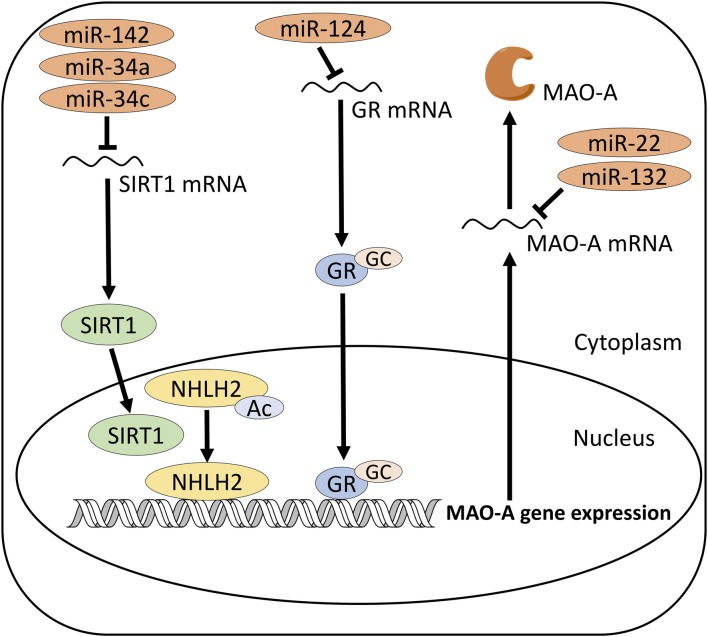
Proposed mechanism of MAO-A regulation by microRNAs (miRNAs). miR-142, 34a and 34c target SIRT1, which activates MAO-A gene expression via deacetylation of NHLH2. miR-124 targets GR, which activates MAO-A gene expression by binding to MAO-A promoter region. miR-22 and 132 may directly target MAO-A mRNA. SIRT1, Sirtuin 1; NHLH2, Nescient Helix-Loop-Helix 2; GC, Glucocorticoid; GR, Glucocorticoid receptor; MAO-A, monoamine oxidase A.

miR-132 is a brain-enriched miRNA, which shows higher expression levels in the brain, compared to other organs in humans and mouse (Sempere et al., 2004), suggesting that this miRNA could be a good target to modulate MAO-A specifically in the brain. miR-132 is a potential miRNA which targets MAO-A gene (Figure 1). *Toxoplasma gondii* infection in human neuroepithelioma cells and mice brain upregulate miR-132, decrease MAO-A gene expression and protein levels and decrease dopamine metabolism but not 5-HT metabolism, suggesting that miR-132 could downregulate MAO-A and selectively affect dopaminergic system rather than 5-HT system (Xiao J. et al., 2014). In this study, however, the interaction between miR-132 and MAO-A gene was validated only with *in silico* prediction. Therefore, experimental validation of their interaction should be the subject of additional studies. Chronic unpredictable mild stress (CUMS) downregulates the levels of miR-132 in the hippocampus but not in the frontal lobe of mice, whereas this effect of CUMS in the hippocampus is reduced by duloxetine treatment (Pan and Liu, 2015), indicating that antidepressant effects of duloxetine might be mediated by miR-132 in the hippocampus.

### miR-34 Family

miR-34 family is highly conserved across species. miR-34a is expressed at the highest levels in the brain among different mouse organs, although it is present at moderate levels in the lung, heart and kidney as well (Bommer et al., 2007). miR-34a targets SIRT1 mRNA (Yamakuchi et al., 2008; Yamakuchi and Lowenstein, 2009; Tarantino et al., 2010; Figure 1), which has two binding sites of miR-34 family (Zovoilis et al., 2011). miR-34c is highly expressed in the hippocampus, compared to other brain regions (Zovoilis et al., 2011). APPPS1-21 mice, a model of amyloid pathology, show an increase in miR-34c levels and a decrease in SIRT1 protein, but not SIRT1 mRNA levels, in the hippocampus, in line with an action of miR-34c to repress the translation of mRNA (Zovoilis et al., 2011). miR-34 seed inhibitor abolishes the reduction of SIRT1 protein and the impaired memory function in APPPS1-21 mice (Zovoilis et al., 2011). Since SIRT1 is involved in the regulatory system of MAO-A in the brain (Libert et al., 2011), miR-34c could be used as a therapeutic target for depression.

Acute restraint stress and chronic social defeat stress upregulate miR-34c in the amygdala of mice (Haramati et al., 2011). miR-34c overexpression leads to a decrease in corticotropin-releasing factor receptor type 1 (CRFR1) expression in the amygdala via binding to 3’UTR of CRFR1 mRNA. Also, miR-34c overexpression decreases in the responsiveness of cells to CRF in mouse neuroblastoma N2a cells expressing CRFR1 but not CRFR2 (Haramati et al., 2011). However, the functional connection between miR-34c and other factors involved in the regulation of MAO-A in depression or stress has not been studied. Considering that miR-34c can affect SIRT1 (Zovoilis et al., 2011; Figure 1), it would be plausible to hypothesize that miR-34c might be involved in the regulation of MAO-A in stress-related disorders.

### miR-124

miR-124, one of the highly conserved miRNAs, is the most abundant miRNA in the brain, which is involved in various pathophysiology in the brain, such as neurodegeneration, stress response, brain tumor and neuroimmune disorders (Reviewed in Sun et al., 2015). miR-124 is a brain-specific miRNA (Babak et al., 2004; Barad et al., 2004; Kim et al., 2004; Sempere et al., 2004; Cao et al., 2006); therefore, modulation of miR-124 is likely to be a good strategy for brain-specific inhibition of MAO-A, which has not been achieved with conventional MAO-A inhibitors. In addition, real-time RT-PCR showed that miR-124 expression levels are higher in the cerebral cortex than cerebellum and spinal cord, suggesting the regional difference in miR-124 expression in the central nervous system (Mishima et al., 2007). The role of miRNA-124 in stress and depression is also well studied. Acute restraint stress decreases miR-124 but increases mineralocorticoid receptors (MRs), a target of miR-124, in the amygdala of mice, indicating the function of miR-124 as a regulator of MR levels (Mannironi et al., 2013). Chronic ultra-mild stress induces the downregulation of miR-124 in the hippocampus of mice, which is abolished by chronic treatment with imipramine (Higuchi et al., 2016). In addition, miR-124 overexpression in the hippocampus leads to stress resilience to chronic ultra-mild stress, while the inhibition of miR-124 increases the stress susceptibility of mice (Higuchi et al., 2016). In another study (Bahi et al., 2014), however, chronic social defeat stress upregulated miR-124 in the hippocampus but not in the cortex of rats. The overexpression of miR-124 in the hippocampus exacerbates depressive behavior; the inhibition shows anti-depressant-like effects. Both overexpression and inhibition of miR-124 in the cortex, however, show no effect on behavior (Bahi et al., 2014). This result indicates that effect of miR-124 is brain-region-specific. The difference in the roles of miR-124 in the hippocampus between these two studies (Bahi et al., 2014; Higuchi et al., 2016) suggests that effects of miR-124 may depend on types of stress, model animals and genetic background.

Chronic corticosterone treatment increases miR-124 in the prefrontal cortex of rats and *in silico* prediction showed that the promoter region of miR-124 has GR binding motif (Dwivedi et al., 2015), suggesting that miR-124 may modulate the response of the prefrontal cortex to hyperactivity of the HPA axis in stress. A recent study suggested that miR-124 targets GR mRNA and miR-124 mimics decreases protein levels of GR in HEK 293 cells (Wang et al., 2017; Figure 1). Inhibition of miR-124 by antagomir abolishes the reduction of the hippocampal GR protein, the decrease of sucrose preference and the increase of immobility time induced by chronic corticosterone administration (Wang et al., 2017), indicating the involvement of miR-124 in these behavioral changes and the antidepressant-like effects of antagomir for miR-124. GC signaling is one of the factors which regulate the expression of MAO-A (Ou et al., 2006); therefore, miR-124 might affect the regulation of MAO-A by changing GR protein levels.

### miR-22

Microarray study showed higher expression levels of miR-22 in the brain tissues of rat, such as the hippocampus, olfactory bulb, brain stem, cortex and hypothalamus, compared to the peripheral organs, including the heart, liver, kidney and lung (Hua et al., 2009), suggesting that miR-22 is brain-enriched miRNA. Two widely used miRNA target prediction, miRanda and TargetScan, show that 3’-UTR of human MAO-A gene has miR-22 target sites. Muiños-Gimeno et al. (2011) investigated the functional relationship between miRNAs and MAO-A gene expression by luciferase assay. miR-22 downregulates MAO-A gene as well as genes related to psychiatric disorders, such as brain-derived neurotrophic factor (BDNF) and 5-HT_2C_ receptor (Muiños-Gimeno et al., 2011). In addition, many studies suggested that miR-22 targets SIRT1 gene in various types of cells, such as glioblastoma, cardiomyocytes, renal cell carcinoma and breast cancer cell lines (Xu et al., 2011; Chen et al., 2016; Du et al., 2016; Kurylowicz et al., 2016; Xiong et al., 2016; Zhang et al., 2016, 2017; Zou et al., 2017). SIRT1 is one of the regulatory factors of MAO-A gene expression, which activates NHLH2 (also known as neuronal SCL-like protein 2, NSCL-2), a transcription activator for MAO-A gene (Libert et al., 2011). Therefore, expression and functions of miR-22 in MAO-A containing cells in the brain should be the subject in future studies (Figure 1).

## Possible Mechanism of MAO-A Regulation by miRNAs

### miRNAs Modulate MAO-A Regulators

miRNAs may change the protein levels of the regulators of MAO-A and indirectly influence MAO-A in the brain. In this type of regulation, miRNAs downregulate the gene transcription of the regulatory factors of MAO-A, which in turn lead to reduced protein levels of the regulatory factors (Figure 1). This could result in upregulation of MAO-A if the regulatory factors are inhibitory in nature, or downregulation of MAO-A if the regulatory factors are activators of MAO-A. For example, miR-142 targets SIRT1, an activator of MAO-A gene expression, and influence MAO-A (Chaudhuri et al., 2013a). SIRT1 is a target of many types of miRNAs (Bicker and Schratt, 2010; Gao et al., 2010; Saunders et al., 2010; Yamakuchi, 2012; Zhou et al., 2012; Ahn et al., 2013; Chaudhuri et al., 2013a; Choi and Kemper, 2013), although miRNA-mediated regulation of SIRT1 in depression has not been fully understood. Previous studies have found several regulatory factors of MAO-A, such as KLF11 (Grunewald et al., 2012; Harris et al., 2015), R1 (Ou et al., 2006; Johnson et al., 2011) and RINES (Kabayama et al., 2013). Except for SIRT1, relationship between miRNAs and these regulatory factors of MAO-A have not been studied, although a few studies show that some miRNAs target several types of KLF family proteins (Kinoshita et al., 2010; Tian et al., 2010; Muiños-Gimeno et al., 2011; Nagata et al., 2014; Xiao H. et al., 2014; Ma et al., 2015; Periyasamy et al., 2018), suggesting a possibility that KLF11 also could be a target of miRNAs in the brain. Elucidating the relationship between miRNAs and these regulatory factors would be useful to find miRNAs which can be novel therapeutic targets for better control of MAO-A in the brain.

### miRNAs Directly Target MAO-A mRNA

Another possible mechanism by which miRNAs changes MAO-A levels is that miRNAs directly target MAO-A mRNA as proposed about miR-132 (Xiao J. et al., 2014; Figure 1) and miR-22 (Muiños-Gimeno et al., 2011; Figure 1). Expression of miRNAs is regulated by transcription factors. Regulatory elements controlling the expression of miRNAs are located within 1 kb upstream of pre-miRNA genes (Lee et al., 2007). So far, transcription factor-miRNA pathways in the regulation of MAO-A have not been identified. Patients with depression and animal models of depression show changes in miRNA expression (Dwivedi, 2011, 2016; Chan and Kocerha, 2012; Ma et al., 2016) as well as the levels of transcription factors, such as KLF11 and R1 (Johnson et al., 2011; Harris et al., 2015). It has been reported that KLF3 and KLF4 regulate the expression levels of miR-182 (Sachdeva et al., 2015; Segura et al., 2017), indicating that KLF family of proteins may regulate the expression levels of miRNAs and further modulate the downstream signaling of the pathway. Therefore, it is plausible to hypothesize that KLF11 could regulate the expression of miRNAs in the brain and influence the regulation of MAO-A. However, the relationship between these transcription factors and miRNAs have not been fully elucidated. Predicting the putative binding sites of transcription factors in the promoter region of pre-miRNA genes and experimentally elucidating the relationship of transcription factors with miRNA expression would be required.

## miRNA Therapeutics for Depression

The techniques to modulate the function of miRNAs in the brain are important issues which may decide whether miRNAs can be potential therapeutic targets for depression.

### Methodologies to Modulate miRNAs

There are several approaches to manipulate miRNAs. miRNA mimics could compensate the downregulation of specific miRNAs observed in the pathogenic state. Introduction of synthetic oligonucleotides that mimic a specific miRNA or introduction of viral vectors that over-express a specific miRNA are the examples of therapeutic approaches by miRNA mimics. Antagomirs are single-stranded RNAs that have complementary sequences to miRNA targets and inhibit the activity of miRNAs (Krützfeldt et al., 2005). Another methodology called “miR sponges” has also been used to modulate the miRNAs (Ebert et al., 2007). Sponge RNAs contain multiple miRNA binding sites to soak up a miRNA of interest in cells and create a loss of function of the miRNA. Another proposed method to modulate the function of miRNA is target protection. Modified antisense oligonucleotides complementary to a miRNA binding site of specific mRNA disrupt the interaction between miRNA and mRNA, thus increasing the stability of mRNA (Choi et al., 2007; Staton and Giraldez, 2011). Designs of miRNA mimics, antagomirs, miR sponges and target protectors are decided based on the sequence of miRNAs or miRNA binding sites in mRNA. The binding of antagomirs and miR sponges with miRNAs are done with the Watson-Crick base paring of nucleotides, which makes designing antagomirs and miR sponges easier, compared to designing/screening molecules which bind to a specific protein.

In experimental settings, these methodologies have been used to modulate the function of miRNAs in the brain. For example, miR-124 antagomir injected into the lateral ventricle shows antidepressant effects in corticosterone-induced depressive-like mice by targeting GR (Wang et al., 2017). miR sponge treatment to primary neurons (Olde Loohuis et al., 2015) and hippocampal injection of lentiviral miR sponge (Bofill-De Ros et al., 2015) modulate miRNAs. Therefore, these methodologies are effective to modulate the function of miRNAs in the brain.

### Drug Delivery in miRNA Therapeutics

Delivery of the reagents targeting miRNAs in the brain is one of the major difficulties in miRNA therapeutics of psychiatric diseases. Reagents for miRNA therapeutics are required to cross the blood-brain barrier to access the miRNAs in the brain. Intravenous administration of antagomir targeting miR-16 efficiently silences the miR-16 in most of the peripheral tissues except the brain (Krützfeldt et al., 2005), indicating that delivery of antagomirs to the brain is one of the challenges in miRNA therapeutics in the central nervous system.

In experimental settings, antagomirs and miRNA sponges have often been injected directly into the brain of animals to test their effects on mRNA targets involved in the pathology in the brain (Jimenez-Mateos et al., 2012, 2015; Bofill-De Ros et al., 2015; Zheng et al., 2016). Intranasal administration of labeled antagomir to Alzheimer’s disease model mice demonstrated that the antagomir reaches the brain and shows similar effects to intraventricular injection of the antagomir (Lee et al., 2012), indicating that intranasal administration of antagomirs might be a possible therapeutic approach. In addition, a study showed that rabies virus glycoprotein-labeled nanomaterials injected intravenously are capable of delivery of miRNA mimics to the brain *in vivo* (Hwang et al., 2011). Many types of delivery system for miRNAs, such as lipid-based carriers, polymer-based carriers and carbon-based carriers have been developed (Reviewed in Wen, 2016); however, most of them have been used to deliver miRNAs to peripheral tissues. Development of a delivery system of miRNAs into the brain would be one of the key problems that we need to overcome for successful miRNA therapeutics of psychiatric diseases.

## Conclusion

Here, we reviewed whether miRNAs can be therapeutic targets for depression. Several studies suggest that miRNAs indirectly regulate MAO-A by targeting the regulators of MAO-A expression, such as SIRT1, indicating the potential of miRNAs as therapeutic targets. No studies have reported a miRNA directly targeting MAO-A mRNA, except for studies that predict that miR-132 and miR-22 could target MAO-A mRNA (Muiños-Gimeno et al., 2011; Xiao J. et al., 2014). Brain-specific (miR-124) or brain-enriched (miR-132, miR-34 and miR-22) miRNAs can be good therapeutic targets to modulate MAO-A specifically in the brain, which could avoid adverse effects caused by conventional MAO-A inhibitors in peripheral organs. More detailed investigation on the localization of miRNAs in the brain and the potential association between transcription factors and miRNAs related to MAO-A regulation would be necessary to further discuss the potential of these miRNAs as therapeutic targets to control MAO-A in specific brain regions related to the pathophysiology of depression.

## Author Contributions

YH and IP designed research. YH wrote the article. IP and TS edited the article.

## Conflict of Interest Statement

The authors declare that the research was conducted in the absence of any commercial or financial relationships that could be construed as a potential conflict of interest.
